# Efficacy and mechanism of mandibular advancement devices for persistent sleep apnea after surgery: a prospective study

**DOI:** 10.1186/s40463-016-0167-x

**Published:** 2016-11-03

**Authors:** Huiping Luo, Xulan Tang, Yuanping Xiong, Lili Meng, Hongliang Yi, Shankai Yin

**Affiliations:** Department of Otolaryngology, the Affiliated Sixth People’s Hospital of Shanghai Jiao Tong University, Shanghai, China

**Keywords:** Persistent sleep apnea, Uvulopalatopharyngoplasty, Mandibular advancement devices, Upper Airway, Computed tomography

## Abstract

**Background:**

To explore the feasibility, the efficacy, and the mechanism of mandibular advancement devices (MAD) in the treatment of persistent sleep apnea after surgery.

**Methods:**

Nineteen patients who failed uvulopalatopharyngoplasty (UPPP) or UPPP plus genioglossus advancement and hyoid myotomy (GAHM) were given a non-adjustable MAD for treatment. All patients had polysomnography (PSG) at least 6 months post-UPPP with and without the MAD. Seventeen patients had computed tomography (CT) examinations.

**Results:**

After the application of MAD, the apnea hypopnea index (AHI) decreased significantly from 41.2 ± 13.1/h to 10.1 ± 5.6/h in the responder group. The response rate was 57.9 % (11/19). During sleep apnea/hypopnea acquired from sedated sleep, the cross-sectional area and anterior-posterior and lateral diameters of the velopharynx enlarged significantly from 4.2 ± 6.0 mm^2^ to 17.5 ± 15.3 mm^2^, 1.9 ± 2.3 mm to 6.5 ± 4.1 mm, and 1.1 ± 1.3 mm to 2.6 ± 2.1 mm, respectively (*P* < 0.01) in the responder group with MAD. The velopharyngeal collapsibility also decreased significantly from 83.3 ± 21.8 % to 46.5 ± 27.1 %. The glossopharyngeal collapsibility decreased from 39.8 ± 39.1 % to −22.9 ± 73.2 % (*P* < 0.05).

**Conclusion:**

MAD can be an effective alternative treatment for patients with moderate and severe OSAHS after surgery. The principal mechanisms underlying the effect of MAD are expansion of the lateral diameter of the velopharynx, the enlargement of the velopharyngeal area, the reduction of velopharyngeal and glossopharyngeal collapsibility, and the stabilization of the upper airway.

## Background

Obstructive sleep apnea hypopnea syndrome (OSAHS) is a common condition that is associated with serious adverse health consequences. Its prevalence is about 2 to 4 % in adults [1]. Continuous positive airway pressure (CPAP) is the preferred method for treating OSAHS; however, 40 % patients with OSAHS cannot tolerate or are unwilling to accept CPAP treatment [2]. They instead choose to undergo other treatments, such as surgery. Uvulopalatopharyngoplasty (UPPP) is the mainstay surgical approach in the treatment of patients with palatopharyngeal obstruction. However, the response rate to this procedure was only 50 % in a short-term follow-up [3]. UPPP plus genioglossus advancement and hyoid myotomy (GAHM) has been applied in patients with OSAHS with palatopharyngeal and tongue base obstructions, but the response rate was about 67 % [4]. The management of non-responders is difficult. Usually, CPAP and more invasive operations such as mandibular and maxillary advancement (MMA) are recommended, but are rarely accepted. Mandibular advancement devices (MAD) are non-invasive, cheap, and usually used in patients with primary snoring or mild OSAHS. The response rate is 77 % [5]. Millman studied 24 patients who failed UPPP with an adjustable MAD [6]. Eighteen patients underwent polysomnography evaluations. Twelve patients had been responders with MAD in place. MAD treatment might be considered for use in non-responders after surgery, but its feasibility and efficacy must be further demonstrated. The mechanism of MAD treatment of sleep apnea is unclear. In this study, 19 non-responders after upper airway surgery were given non-adjustable MAD. The feasibility, the efficacy, and the mechanism of MAD in the treatment of persistent sleep apnea after surgery were evaluated.

## Methods

### Subjects

The inclusive criteria are: 1. Subjects accepted PSG examination at least 6 months after operation, and post-operation moderate or severe OSAHS were diagnosed. 2. Patients wanted to accept therapy but refused to accept CPAP or reoperation. 3. Patients without occlusal lower respiratory disorders, periodontal disease or the temporomandibular joint (TMJ) diseases. The exclusive criteria are: 1. Patients with central sleep apnea. 2. Patients with severe respiratory disorders. 3. Patients with nasal obstructive diseases. 4. The maximal jaw forward distance is less than 6 mm. 5. Patients with the alveolar bone resorption. 6. The crown root ratio is over 2:1. 111 post-operation OSAHS patients were followed up, only 19 patients (17 men, 2 women) with four surgical procedures met the inclusive criteria, which were Han-uvulopalatopharyngoplasty (HUPPP) (9), HUPPP + GAHM (1), Z-palatopharyngoplasty (ZPPP) [5] (2), and ZPPP + GAHM (7). Subjects with persistent OSAHS had an average age of 42.4 years (23–58 years), average body mass index (BMI) of 28.2 kg/m^2^ (21.2–33 kg/m^2^), and average apnea hypopnea index (AHI) of 44.2/h (20.5–83.7/h).

All patients were given non-adjustable MAD called Snore Guards after polysomnographic recordings and investigation of Epworth sleepiness scale (ESS) with an average of 28.6 months after the surgical procedures. Six months after the application of MAD, all patients again underwent all investigations described above. The efficacy and the side effects of MAD were also judged subjectively by a questionnaire before the last sleep study, including the sleep quality of the previous night, patient satisfaction with the device, and side effects.

To determine the mechanism of MAD and the morphological changes in the upper airway that result from its application, computed tomography (CT) of the upper airway was conducted during a daytime nap induced by intravenous midazolam [7, 8] with and without MAD in place.

### Polysomnography

The sleep studies were carried out in the Sleep Disorders Center of the Affiliated Sixth People’s Hospital of Shanghai Jiao Tong University. Apnea was defined as the total cessation of airflow that was >10 s. Hypopnea was defined as an event that met two of the following three criteria: (1) a decrease in airflow of >50 %; (2) an EEG arousal as defined by the American Sleep Disorders Association scoring criteria; or (3) oxygen desaturation of >3 %. OSAHS was defined as an AHI of more than five episodes per hour during sleep in patients with symptoms of the disorder [9]. Therapeutically effective is defined as a decrease in AHI of >50 % of that before treatment with MAD in place. Treatment success was defined as AHI <5/h with MAD in place [10].

### Upper airway CT

Upper airway CT (Lightspeed VCT, GE, Finland) was performed in seventeen patients (two patients didn’t accept CT examination because of personal reasons) before and after MAD application under three conditions: quiet breathing, the end of deep inspiration, and apnea. The time of CT acquisition is about one breathing cycle. All CT images were reconstructed from the middle sagittal planes. The planes of the upper airway at the level of the nasopharynx (the extension cord over the lower edge of the hard palate), the velopharynx (the tip of the uvula), the glossopharynx (the narrowest part of the glossopharynx), and the epiglotopharynx (5-mm below the tip of the epiglottis) were obtained during the phase of quiet tidal breathing, end of deep inspiration, and sleep apnea/hypopnea. The cross-sectional area, anterior-posterior diameter, and lateral diameter of each plane of the upper airway were manually measured by using electronic calipers by the same technician. The degree of collapse was calculated as follows: (quiet breathing cross-sectional area at the end of deep breathing or sleep apnea) / cross-sectional area during quiet breathing [9]. CT scans were evaluated by the same investigator who was blinded to both responders and non-responders. We informed all patients about the purposes of the CT examination, as well as the short-term potential hazards or long-term complications caused by frequent radiation examinations, after which all patients signed an informed consent. With 55 months of follow-up, no side effects or adverse reactions were seen to be from radiation exposure.

### MAD

Before the intervention of MAD, a clinical examination of the stomatognathic system, which included measurement of mandibular mobility, palpation of the temporomandibular joints and masticatory muscles, and recording of pain when the mandibular moved forward, was performed. In the present study, the same dentist took care of all patients, and the same dental technician was responsible for the preparation of all appliances.

**Fig. 1:**
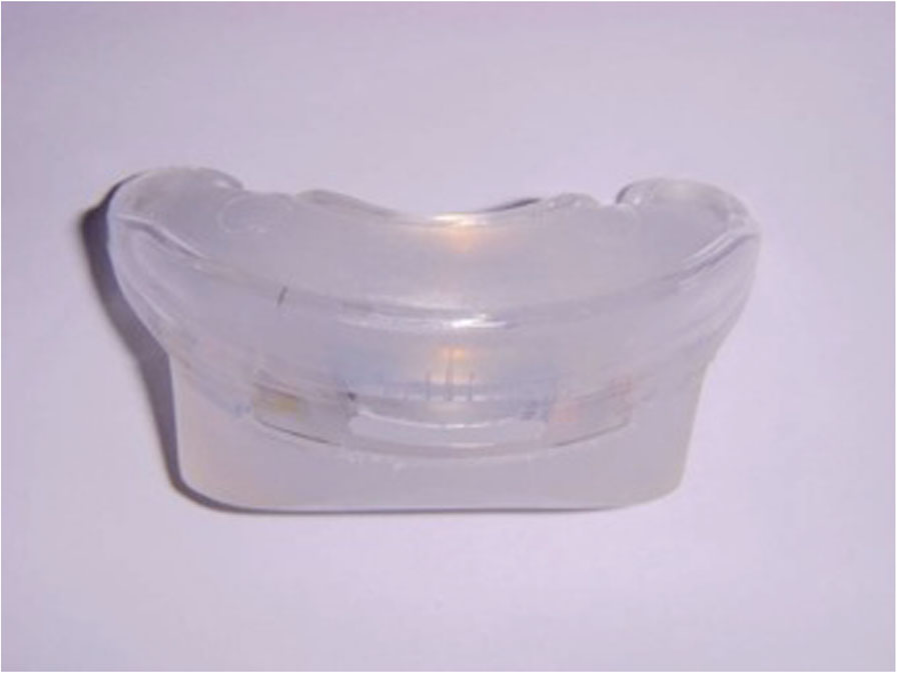
Snore Guard XT-1B (produced by Xintai Company, Peking)

**Fig. 2:**
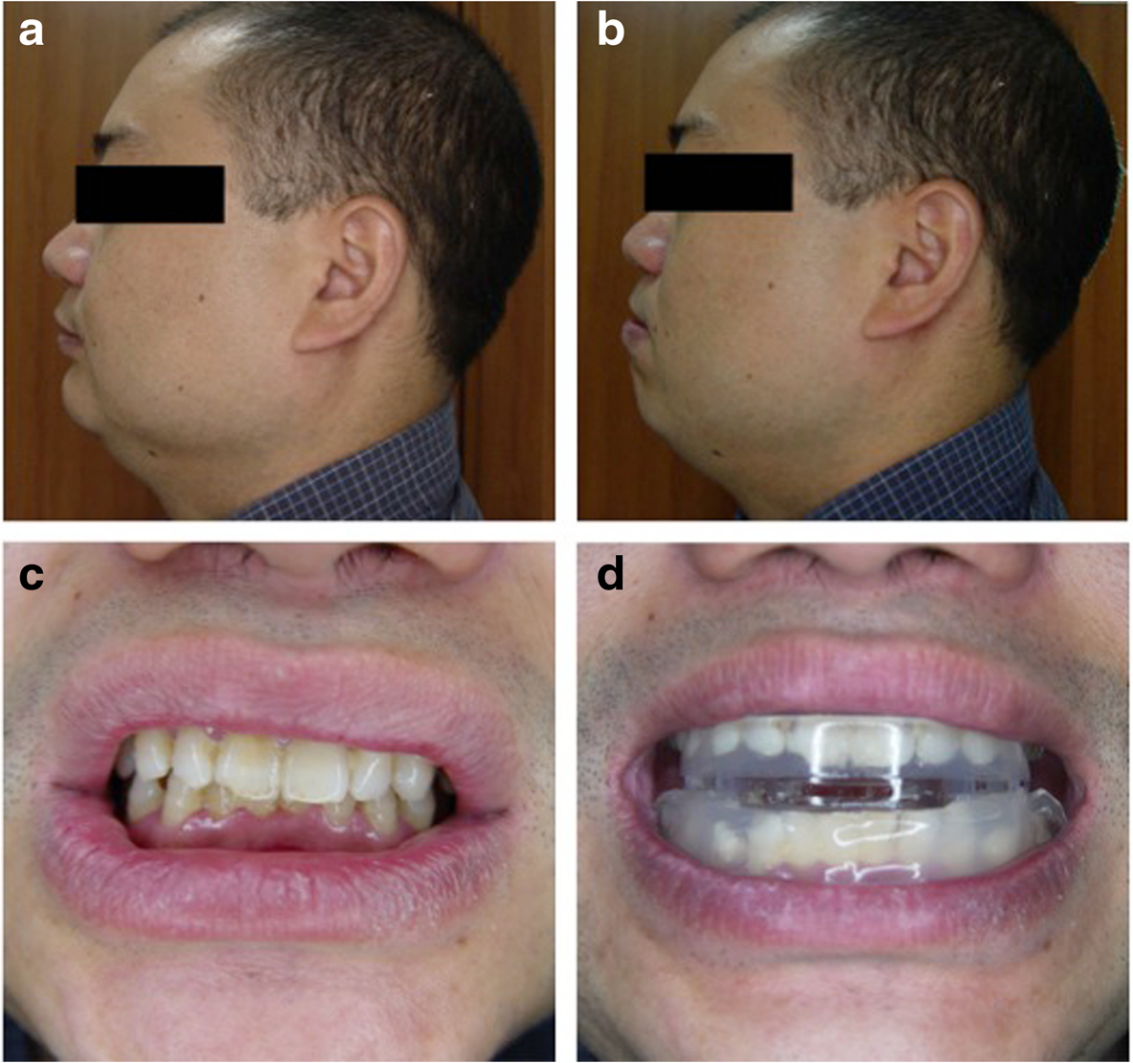
Lateral view before wearing an MAD (**a**), Lateral view after wearing an MAD (**b**), Front view before wearing an MAD (**c**), Front view after wearing an MAD (**d**)

We used a MAD called ‘Snore Guard’ (Xintai Ltd., Beijing) for the advancement of the mandible (Figs. 1 and 2). The degree of mandibular advancement induced by MAD was adjusted according to the anatomy and tolerance of the individual patient. Patients tried only one position during therapy. In former studies [11–18], physicians measured the maximum distance of mandibular advancement, then choose the position which is about 75 % of the maximum distance and did the MAD moulding at the position following the product instruction. The goal was to minimize nocturnal apnea and snoring while maintaining a comfortable fit. In our study, the mandibular protrusive maneuver of each subject was measured. The averaged distances were 5 to 7 mm, which varied from 51 to 100 % of patients’ maximum protrusive maneuver. After the last fitting, patients were asked to wear the device every night during sleep. The patients were encouraged to wear the device regularly by telephone as well as when they returned for visits at our office [11–14].

### Statistical analysis

Statistical analysis was performed using SPSS version 18.0. The paired-samples *t*-test was used to compare variables of PSG results and CT parameters pre- and post-treatment with MAD. One-way analysis of variance (ANOVA) was used for comparisons of PSG results and CT parameters between the responder and non-responder groups. All parametric results were expressed as means ± standard deviation (SD). A statistically significant difference was defined as *P* < 0.05 for all parameters.

## Results

### Polysomnographic results

All-night polysomnographic data of 19 patients were available. Patients were grouped into responders and non-responders according to criteria described in the Method portion. The AHI in both groups was 44.2 ± 19.2/h, and the LSaO_2_ was 77.2 % ± 7.6 % post-surgically; i.e., before the application of MAD. The AHI decreased significantly to 28.2 ± 24.6/h and the LSaO_2_ increased to 79.3 ± 10.4 % with MAD (Table 1). While in the responder group, the AHI decreased significantly from 41.2 ± 13.1/h to 10.1 ± 5.6/h, and the LSaO_2_ increased significantly from 78.1 ± 8.9 % to 85.4 ± 6.5 % with MAD (Tables 2 and 3).

**Table 1:** Body measurement data and ESS and PSG parameters before and after MAD treatment

Parameters	Before MAD	After MAD	*P* value
BMI (kg/m^2^)	28.2 ± 3.2	26.9 ± 7.0	0.373
ESS	8.6 ± 6.2	6.1 ± 6.1	0.025
(S3 + S4)%	12.9 ± 6.5	14.3 ± 8.7	0.459
AHI (/h)	44.2 ± 19.2	28.2 ± 24.6	0.005
LSaO_2_ (%)	77.2 ± 7.6	79.3 ± 10.4	0.327

*Abbreviations*: *BMI* average body mass index, *ESS* epworth sleepiness scale, *(S3 + S4)%* the percentage of stage 3 and stage 4 sleep (deep sleep) in the total sleep time, *AHI* apnea hypopnea index, *LSaO2* lowest oxygen saturation

Eleven patients were classified as responders,two of whom were complete response with MAD as shown by an AHI of less than five, and eight as non-responders; the response rate was 57.9 % (11/19). The ESS decreased from 8.6 to 6.1 after the application of MAD. None of the patients had a substantial change in body measurements during the study (Tables 2 and 3).

**Table 2:** Body measurements, ESS results between responders and non-responders before treatment

Parameters	Responders	Non-responders	*P* value
BMI(kg/m^2^)	27.9 ± 3.3	28.5 ± 3.3	0.709
Neck circumference (cm)	41.6 ± 1.9	40.0 ± 3.2	0.199
Waist circumference (cm)	100.6 ± 7.4	99.7 ± 8.8	0.804
Hip circumference (cm)	104.0 ± 6.9	104.5 ± 6.2	0.860
Waist-to-hip ratio	1.0 ± 0.1	1.0 ± 0.1	0.578
ESS	7.4 ± 6.1	9.9 ± 6.4	0.401
AHI (/h)	41.2 ± 13.1	47.6 ± 24.8	0.487
LSaO_2_ (%)	78.1 ± 8.9	76.2 ± 6.1	0.604

*Abbreviations*: *BMI* average body mass index, *ESS* Epworth sleepiness scale, *AHI* apnea hypopnea index, *LSaO2* lowest oxygen saturation

**Table 3:** Body measurements, ESS results between responders and non-responders after treatment

Parameters	Responders	Non-responders	*P* value
BMI(kg/m^2^)	27.6 ± 2.4	26.2 ± 10.1	0.674
Neck circumference (cm)	40.9 ± 1.6	40.1 ± 3.1	0.482
Waist circumference (cm)	96.7 ± 7.5	100.1 ± 9.3	0.408
Hip circumference (cm)	100.6 ± 4.5	103.1 ± 5.0	0.271
Waist-to-hip ratio	1.0 ± 0.1	1.0 ± 0.1	0.904
ESS	5.4 ± 5.8	6.8 ± 6.6	0.634
AHI (/h)	10.1 ± 5.6	48.4 ± 21.5	0.000
LSaO_2_ (%)	85.4 ± 6.5	72.6 ± 10.0	0.004

*Abbreviations*: *BMI* average body mass index, *ESS* Epworth sleepiness scale, *AHI* apnea hypopnea index,*LSaO2* Lowest oxygen saturation

### Upper airway CT results

**Fig. 3﻿:**
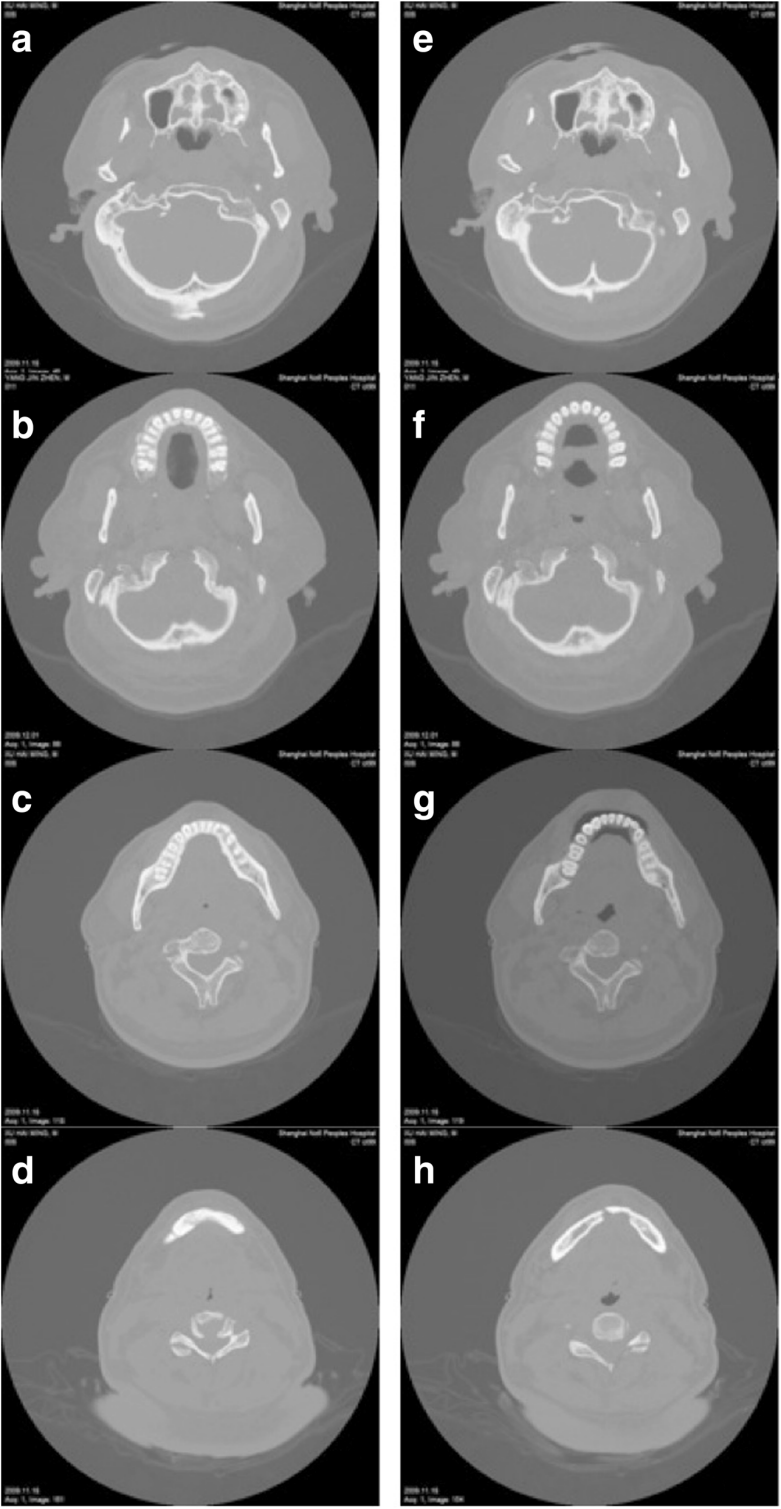
CT scan of responders under the condition of sleep apnea. Without OA in place: nasopharynx (**a**), velopharynx (**b**), glossopharynx (**c**), epiglotopharynx (**d**). With OA in place: nasopharynx (**e**), velopharynx (**f**), glossopharynx (**g**), epiglotopharynx (**h**)

In this study, cephalometric examinations were taken both without and with MAD in place, but the result showed no significant difference. As a three-dimensional imaging examination, CT showed more accurate changes in the upper airway (Fig. 3). Upper airway CT was performed in 17 patients before and after the MAD application (Table 4). In non-responders, the change was not significance before and after the MAD application, which is not explained in text.

**Table 4:** Upper airway CT results of responders (*n* = 9) (mm^2^)

Respiratory status	Parameters	Plane	Before MAD	After MAD	*P* value
Quiet breathing	CSA	NP	131.0 ± 115.4	185.3 ± 100.5	0.052
		VP	26.8 ± 19.0	46.4 ± 26.4	0.023
		GP	103.8 ± 66.9	93.9 ± 50.8	0.533
		EP	52.0 ± 26.5	38.5 ± 22.1	0.068
	A-P diameter	NP	7.7 ± 4.6	9.3 ± 3.5	0.125
		VP	2.8 ± 1.6	3.6 ± 1.6	0.181
		GP	14.6 ± 5.5	13.9 ± 4.5	0.966
		EP	6.6 ± 2.7	5.5 ± 1.7	0.202
	Lateral diameter	NP	11.8 ± 6.6	16.5 ± 5.1	0.045
		VP	9.7 ± 6.3	12.4 ± 4.7	0.008
		GP	14.6 ± 5.5	13.9 ± 4.5	0.497
		EP	15.1 ± 15.2	8.5 ± 4.2	0.236
Deep inspiration	CSA	NP	197.3 ± 173.9	205.7 ± 183.1	0.375
		VP	18.3 ± 16.3	43.0 ± 37.8	0.186
		GP	131.0 ± 3.6	356.0 ± 189.1	0.172
		EP	39.3 ± 27.4	83.0 ± 37.4	0.195
	A-P diameter	NP	9.3 ± 8.1	9.8 ± 8.7	0.423
		VP	1.7 ± 2.0	2.4 ± 2.2	0.277
		GP	8.1 ± 1.4	13.8 ± 7.9	0.264
		EP	5.4 ± 1.0	8.9 ± 1.7	0.145
	Lateral diameter	NP	14.6 ± 12.9	15.1 ± 13.6	0.580
		VP	6.7 ± 7.7	11.6 ± 10.7	0.199
		GP	19.0 ± 2.5	28.5 ± 7.4	0.197
		EP	9.1 ± 7.3	10.6 ± 5.9	0.537
Sleep apnea /hypopnea	CSA	NP	133.8 ± 109.3	163.9 ± 94.5	0.082
		VP	5.0 ± 6.4	21.2 ± 10.5	0.000
		GP	77.0 ± 68.6	86.0 ± 51.3	0.684
		EP	87.4 ± 84.5	66.4 ± 50.4	0.533
	A-A-P diameter	NP	7.1 ± 4.6	8.7 ± 3.7	0.171
		VP	1.3 ± 1.2	2.9 ± 0.9	0.001
		GP	8.9 ± 6.8	9.3 ± 4.5	0.813
		EP	10.2 ± 6.7	8.5 ± 4.1	0.442
	Lateral diameter	NP	12.0 ± 6.7	15.7 ± 6.1	0.080
		VP	2.2 ± 2.3	8.2 ± 3.8	0.001
		GP	9.6 ± 7.8	11.5 ± 4.4	0.427
		EP	9.4 ± 6.9	9.3 ± 5.2	0.985

*Abbreviations*: *CSA* cross-sectional area, *A-P* anterior-posterior diameter, *NP* nasopharynx, *VP* velopharynx,*GP* glossopharynx, *EP* epiglopharynx

During the phase of quiet tidal breathing, the velopharyngeal cross-sectional area and the lateral diameters of the velopharynx and nasopharynx were much larger with MAD in place than without MAD in the responder group (46.4 ± 26.4 mm^2^ vs. 26.8 ± 19.0 mm^2^, 12.4 ± 4.7 mm vs. 9.7 ± 6.3 mm, and 16.5 ± 5.1 mm vs.11.8 ± 6.6 mm, respectively; *P* < 0.05).

During the state of deep inspiration, the changes in the upper airway did not reach statistical significance in both the responder and non-responder groups.

During sleep apnea/hypopnea, the cross-sectional area, anterior-posterior diameter, and lateral diameter of the velopharynx were significantly enlarged at 5.0 ± 6.4 mm^2^ to 21.2 ± 10.5 mm^2^, 1.3 ± 1.2 mm to 2.9 ± 0.9 mm, and 2.2 ± 2.3 mm to 8.2 ± 3.8 mm, respectively (*P* < 0.01) in the responder group with MAD in place (Table 4). The velopharyngeal collapsibility of the responder group decreased significantly from 83.3 ± 21.8 % to 46.5 ± 27.1 % with MAD in place (*P* < 0.01), and glossopharyngeal collapsibility decreased from 39.8 ± 39.1 % to −22.9 ± 73.2 % (*P* < 0.05) (Table 5).

**Table 5:** Collapsibility of the responder group before and after MAD treatment (%)

Respiratory status	Plane	Before MAD	After MAD	*P* value
Deep inspiration	NP	50.9 ± 69.4	47.5 ± 74.3	0.500
	VP	65.7 ± 48.5	6.5 ± 132.3	0.500
	GP	10.2 ± 28.1	−187.2 ± 26.4	0.123
	EP	20.6 ± 44.5	−72.3 ± 8.5	0.244
Sleep apnea /hypopnea	NP	7.4 ± 91.2	−20.0 ± 101.3	0.591
	VP	83.3 ± 21.8	46.5 ± 27.1	0.005
	GP	39.8 ± 39.1	−22.9 ± 73.2	0.031
	EP	−41.6 ± 101.6	−150.1 ± 176.9	0.066

*Abbreviations*: *A-P* anterior-posterior diameter, *NP* nasopharynx, *VP* velopharynx, *GP* glossopharynx, *EP* epiglopharynx

### Side effects

After wearing MAD all night for 1 week, various side effects of different degrees occurred in all patients, including weak occlusion (1/19), excessive saliva (6/19), dry mouth (2/19), temporomandibular joint aches (1/19), dental soreness (9/19), toothache (1/19), loose lower incisors (2/19), mucosal ulceration (2/19), and masseter muscle pain (3/19). However, all patients were able to continue the treatment, and all symptoms were relieved in 1 month with timely and appropriate care.

### Compliance

With reference to the definition of CPAP compliance [15], we considered wearing the MAD for >70 % of nights and wearing it for >4 h per night as good MAD treatment compliance. In this study, 63.2 % (12/19) of patients had good compliance; in fact, these patients wore the MAD all night. Among the patients with poor compliance, three wore the MAD <3 days per week because of loose lower incisors in two patients after UPPP (HUPPP or ZPPP) combined with GAHM, and gum pain caused by MAD deformation in one patient. One patient wore the MAD for <4 h per night because of dry mouth. The survey results had no difference between pre- and post-MAD treatment for both responders and non-responders. The pre- and post-MAD treatment ESS scores of responders were 7.4 ± 6.1 and 5.4 ± 5.8 (*P* = 0.215), and the quality of life scores were 5.5 ± 1.5 and 5.6 ± 1.7 (*P* = 0.772). There was no difference before and after treatment, which maybe related with the low compliance of MAD at home.

## Discussion

MADs are the most commonly used oral appliances and recommended in the treatment of snoring and mild to moderate OSAHS patients, but they were rarely used in patients with severe OSAHS, especially in those with persistent OSAHS condition after surgical treatment. There are multiple MAD designs. Physicians used those devices intending to protrude the mandible, increase tension of soft tissues as the soft palate, lateral pharyngeal walls and the tongue, and improve the airway patency [16]. Many studies were done in past decades, but the mechanism underlying the efficacy of MAD for the treatment of OSAHS was still controversial in terms of whether it helped enlarge the volume of the pharynx cavity or increased the airway stability. Zhao et al. used MAD to treat 11 patients with moderate to severe OSAHS without surgery [12]. In their study, magnetic resonance imaging (MRI) scans were performed at each time when the lower jaw were moved forward by 0, 2, 4, 6, or 8 mm while patients were wakeful or during sleep apnea/hypopnea. They found that during sleep apnea/hypopnea, the velopharyngeal cross-sectional area with MAD in place was larger than that without MAD, especially the coronal axis. No statistically significant changes in sagittal diameter were found between the various mandibular advancements. In our study, the velopharyngeal cross-sectional area, the coronal axis, and the sagittal diameter with MAD in place were all larger than those without MAD, and the difference was statistically significant. Upper airway CT imaging showed that the velopharyngeal cross-sectional area and lateral diameter of the velopharynx were significantly enlarged in the MAD responders, but not non-responders, during quiet tidal breathing and sleep apnea/hypopnea. The result was consistent with previous reports [13, 17, 18].

According to Schwartz *et al*’s study [19], a negative pressure of about −13 cm H_2_O under normal circumstances could cause airway collapse. Huang et al. [20] also measured airway pressures in patients with OSAHS after various treatments. The pressure was −21 cm H_2_O with MAD in place, −18 cm H_2_O after UPPP, and −17 cm H_2_O after the soft palate implant surgery. These results showed that the airway would not collapse as easily if the patients were wearing a MAD. In our study, The velopharyngeal and the glossopharyngeal collapsibility of the responder group was significantly decreased from 83.3 ± 21.8 % to 46.5 ± 27.1 % and from 39.8 ± 39.1 % to −22.9 ± 73.2 %, respectively (*P* < 0.05). These results are consistent with those of previous studies too [13, 17, 20–22].

The results of our study showed that MAD could expand the velopharyngeal coronal axis, enlarge the velopharyngeal cross-sectional area by mandibular advancement, and reduce velopharyngeal and glossopharyngeal collapsibility, resulting in stabilization of the upper airway. During the phase of quiet tidal breathing, the cross-sectional area of nasopharyngeal in the responder group without MAD in place were less than the non-responder group. In further study, we would do some research to validate whether it could be used as a predictor of respond on MAD.

Thus, MAD treatment might be considered for patients with persistent VP or GP, especially VP obstruction after surgery if they are unable to tolerate CPAP and refuse more invasive surgery. Thus, MAD might be an effective and feasible treatment for non-responding patients after upper airway surgery.

The small number of patients and lack of a control group or randomization probably decrease the power of the study. The multiple different pharyngeal and hypopharygneal procedures that were performed decrease the internal and external validity of the study. In our study, we followed 111 post-operational patients in total, and only 19 patients matched the inclusion criteria. Because of the limited number of patients and four types different surgical procedures, we were not able to separate all subjects into groups. Therefore, the correlation between specific surgeries and changes on CT imaging could haven’t been investigated. We have compared the physical examination results and AHIs of both responders and non-responders, and the results showed no significant different. We will conduct a blinded, controlled, larger prospective study, try to include more subjects and use adjustable MAD in the treatment. Hopefully better efficacy could be seen.

## Conclusions

Post-operational MAD treatment can be an effective alternative for patients with moderate and severe OSAHS after surgery. The principal mechanisms underlying the effect of MAD treatment are the expansion of the lateral diameter of the velopharynx, the enlargement of the velopharyngeal area, the reduction of the velopharyngeal and glossopharyngeal collapsibility, and the stabilization of the upper airway.

## Abbreviations

AHI: The apnea hypopnea index
ANOVA: One-way analysis of variance
A-P: Anterior-posterior diameter
BMI: Average body mass index
CPAP: Continuous positive airway pressure
CSA: Cross-sectional area
CT: Computed tomography
EP: Epiglopharynx
ESS: Epworth sleepiness scale
GAHM: Genioglossus advancement and hyoid myotomy
GP: Glossopharynx
MAD: Mandibular advancement devices
MMA: Mandibular and maxillary advancement
NP: Nasopharynx
OSAHS: Obstructive sleep apnea hypopnea syndrome
PSG: Polysomnography
SD: Standard deviation
TMJ: The temporomandibular joint
UPPP: Uvulopalatopharyngoplasty
VP: Velopharynx
ZPPP: Z-palatopharyngoplasty
